# Factors affecting the recurrence of acute cholecystitis after treatment with percutaneous cholecystostomy

**DOI:** 10.1186/s12893-023-02042-2

**Published:** 2023-05-25

**Authors:** Korhan Tuncer, Gizem Kilinc Tuncer, Bülent Çalık

**Affiliations:** 1 Department of General Surgery, University of Bakırçay, Çiğli Training and Research Hospital, Izmir, Turkey; 2 Department of General Surgery, University of Health Sciences Izmir Bozyaka Training and Research Hospital, Izmir, Turkey

**Keywords:** Acute cholecystitis, Percutaneous cholecystostomy, Recurrence, Tokyo guidelines, Calculous cholecystitis

## Abstract

**Background:**

The aim of this study was to determine the recurrence rate of patients who did not have interval cholecystectomy after treatment with percutaneous cholecystostomy and to investigate the factors that may affect the recurrence.

**Methods:**

Patients who did not undergo interval cholecystectomy after percutaneous cholecystostomy treatment between 2015 and 2021 were retrospectively screened for recurrence.

**Results:**

36.3% of the patients had recurrence. Recurrence was found more frequently in patients with fever symptoms at the time of admission to the emergency department (p = 0.003). Recurrence was found to be more frequent in those who had a previous cholecystitis attack (p = 0.016). It was determined that patients with high lipase and procalcitonin levels had statistically more frequent attacks (p = 0.043, p = 0.003). It was observed that the duration of catheter insertion was longer in patients who had relapses (p = 0.019). The cut-off value for lipase was calculated as 15.5, and the cut-off value for procalcitonin as 0.955, in order to identify patients at high risk for recurrence. In the multivariate analysis for the development of recurrence, presence of fever, a history of previous cholecystitis attack, lipase value higher than 15.5 and procalcitonin value higher than 0.955 were found to be risk factors.

**Conclusions:**

Percutaneous cholecystostomy is an effective treatment method in acute cholecystitis. Insertion of the catheter within the first 24 h may reduce the recurrence rate. Recurrence is more common in the first 3 months following removal of the cholecystostomy catheter. Having a previous history of cholecystitis attack, fever symptom at the time of admission, elevated lipase and procalcitonin are risk factors for recurrence.

## Introduction

Acute cholecystitis (AC) is an inflammatory process of the gallbladder that usually occurs due to gallstones. It is also one of the most common emergency applications. Its treatment is surgical and laparoscopic cholecystectomy is accepted as the standard treatment method today. However, in patients with high surgical risk, different treatments have been sought to reduce surgery-related complications and mortality. Gallbladder drainage method with percutaneous cholecystostomy (PC) was reported by Radder [1] in 1980. The PC method first served as a bridge treatment for interval cholecystectomy in patients who were not suitable for surgery [2]. However, as the surgically unsuitable and aging population increases, the PC method is used more and more [3]. However, there is insufficient evidence that it can be used as a definitive treatment for AC.

PC is a procedure with a high success rate (85.6%) and low mortality (0.36%). There is a mortality rate of 0.96% in interval cholecystectomy after PC and treatment with antibiotics of septic cholecystitis [4]. However, interval cholecystectomy is not performed in every patient after PC. Studies have shown that the recurrence rate of AC after PC treatment varies between 4% and 40% [5–7]. Determining the factors predicting the prognosis after treatment with PC is important to evaluate the risk of recurrence.

The aim of this study is to determine the recurrence rate of patients who do not have interval cholecystectomy after treatment with PC and to investigate the factors that may affect recurrence.

## Methods

### Patients and ethical approval

Patients who were diagnosed with AC on admission to the emergency department between January 2015–2021 and treated by inserting a PC according to the Tokyo Guidelines 2018 (TG18) were included in the study [8, 9]. Patients whose data were missing or were not followed up regularly were excluded in the study. Patients who were under 18 years of age, operated within the first year following the catheter extraction, also had choledocholithiasis, abscess secondary to AC, or hepatopancreatobiliary system malignancy were excluded in the study.

This thesis study was carried out after the approval of the Health Sciences University Izmir Tepecik Training and Research Hospital Ethics Committee (Approval no:2021/05–12). The study made in accordance with the Helsinki Declaration. This study was registered on clinicaltrials.gov (ID:NCT05525442).

**Fig. 1:**
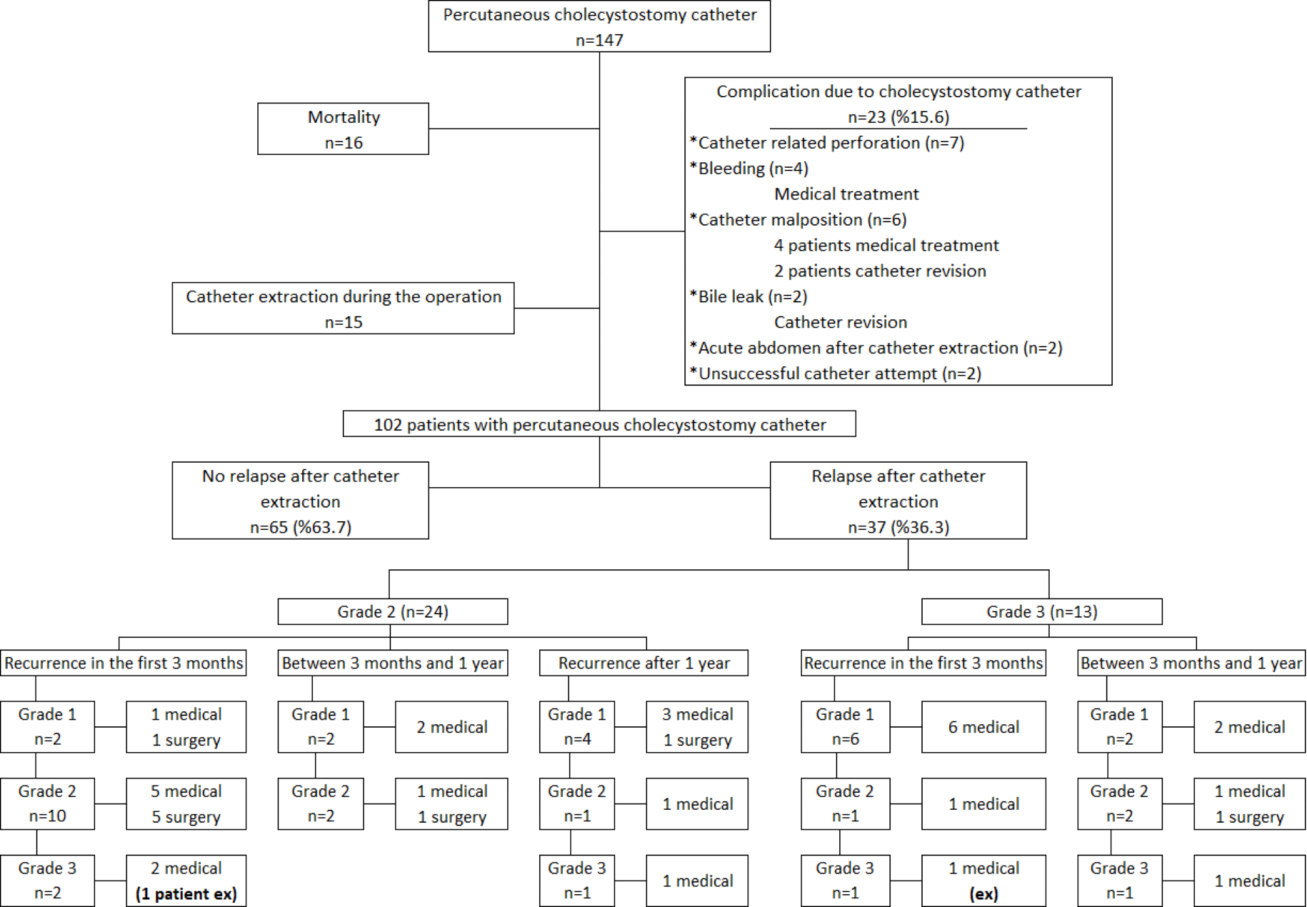
Flow chart of the study

All catheters were placed under local anesthesia. An 8–10 French pigtail catheter was inserted transhepatically under fluoroscopic examination by interventional radiologists. All patients were followed up by general surgeons in outpatient clinics after their discharge. Removal of the catheter was decided according to the clinical status of the patient and the daily fluid volume of the catheter. Cholangiography was performed by the radiologists before the cholecystostomy catheter removal. Patients who underwent catheter removal were followed up for recurrence. Patients who had relapses were divided into subgroups according to the time of recurrence. Recurrent cholecystitis episodes were grouped and treated according to the TG18. The flow chart of the study is shown in Fig. 1.

### Statistical analysis

Statistical analyzes were performed using SPSS version 25.0. The conformity of the variables to the normal distribution was examined using analytical methods (Kolmogorov-Smirnov/ShapiroWilk tests). Descriptive analyzes were given using the mean ± standard deviation for normally distributed variables, and the median (Q1-Q3) for non-normally distributed variables. Descriptive statistics were made by giving demographic characteristics, frequency and percentage values.

In order to compare binary groups such as the presence of recurrence in continuous data, the t-test was used in independent groups in those with normal distribution, and the Mann-Whitney U test in those that were not normally distributed. Pearson’s Chi-Square or Fisher’s Exact Chi-Square test was used in the analysis of categorical data. Spearman correlation analysis was used to measure the cross-sectional relationships between related variables and recurrence. In patients with recurrence, subgroup analysis was performed with the same methods. A Kaplan-Meier survival curve for time to disease relapse was also constructed. ROC analysis was used to determine the prognostic capacity of lipase and procalcitonin in the detection of recurrence. Multivariate logistic regression analysis was performed by determining independent factors. Odds-ratio and 95% confidence intervals for each variable were calculated. p < 0.05 was considered statistically significant.

## Results

A total of 147 patients hospitalized with the diagnosis of AC and treated with PC. Only 102 of the patients who met the criteria were included in the study (Fig. 1). The overall complication rate in 147 patients with PC was 15.6% (n = 23), and there was no catheter-related mortality. The cholecystostomy catheter was removed during the operation in 15 patients who were followed up.

The mean age of the 102 patients included in the study was 69.1 ± 15.2 years and 57.8% were male. The median Charlson comorbidity index (CCI) score of the patients was 4. During the follow-up of the patients, 37 (36.3%) patients had recurrence. There was no significant difference in demographic data between patients who had and did not have a recurrence. 18 (17.6%) of the patients had fever at the time of admission and recurrence was common in patients with fever symptoms (p = 0.003). Recurrence was higher in patients who had a previous cholecystitis attack (p = 0.016). Patients who did not have relapses were followed for at least 1-year, and the median follow-up period was 940 days. The comparison of demographic and clinical characteristics of the patients and laboratory findings were shown in Table 1.

**Table 1:** Demographic and laboratory parameters

	All patients	No recurrence	Recurrence	
	n = 102	n = 65	n = 37	p value
Age, mean ± SD	69.1 ± 15.2	68.3 ± 14.3	70.5 ± 16.7	0.496
Gender, n (%)				0.316
Male	59 (57.8)	40 (61.5)	19 (51.4)	
Female	43 (42.2)	25 (38.5)	18 (48.6)	
Comorbidities, n (%)				
Diabetes mellitus	38 (37.3)	22 (33.8)	16 (43.2)	0.345
Hypertension	48 (47.1)	27 (41.5)	21 (56.8)	0.139
Chronic obstructive pulmonary disease	17 (16.7)	10 (15.4)	7 (18.9)	0.645
Chronic heart disease	13 (12.7)	9 (13.8)	4 (10.8)	0.765*
Coronary artery disease	23 (22.5)	12 (18.5)	11 (29.7)	0.190
Cerebrovasculer disease	20 (19.6)	13 (20)	7 (18.9)	0.895
Chronic kidney disease	4 (3.9)	4 (6.2)	0	0.294*
Charlson comorbidity index, median (Q1-Q3)	4 (3–5)	4 (3–5)	4 (3–5)	0.832
Presence of fever, n (%)	18 (17.6)	6 (9.2)	12 (32.4)	**0.003**
Pancreatitis, n (%)	4 (3.9)	2 (3.1)	2 (5.4)	0.620*
History of cholecystitis, n (%)	18 (17.6)	7 (10.8)	11 (29.7)	**0.016**
ERCP, n (%)	8 (7.8)	3 (4.6)	5 (13.5)	0.135*
Follow-up time (days), median (Q1-Q3)	519 (136.8–1109)	940 (519–1366)	58 (27-225.5)	**< 0.001**
Laboratory parameters				
WBC, mean ± SD	16,485 ± 5823	16,114 ± 5945	17,138 ± 5623	0.396
Neutrophil, mean ± SD	13,289 ± 5882	12,958 ± 5983	13,870 ± 5734	0.454
Lymphocyte, median (Q1-Q3)	1200 (900–1700)	1200 (850–1600)	1200 (900–2050)	0.167
Platelet, mean ± SD	264,250 ± 92,110	261,910 ± 86,771	268,380 ± 101,932	0.735
NLR, median (Q1-Q3)	10.2 (6-18.1)	10.3 (6.2–18)	9.6 (5.8–18.3)	0.674
PLR, median (Q1-Q3)	207.2 (148-305.7)	213 (167.4-323.4)	185 (133.4-302.3)	0.229
Hemoglobin, mean ± SD	12.4 ± 1.9	12.5 ± 2	12.4 ± 1.7	0.814
Hematocrit, mean ± SD	37.5 ± 5.5	37.6 ± 5.9	37.1 ± 4.9	0.659
Glucose, median (Q1-Q3)	138 (110.5-178.8)	133 (111.5–168)	144 (108.5–196)	0.400
Creatine, median (Q1-Q3)	1 (0.9–1.3)	1 (0.9–1.2)	1.1 (0.9–1.4)	0.203
Albumin, mean ± SD	3.2 ± 0.6	3.2 ± 0.5	3.2 ± 0.6	0.885
AST, median (Q1-Q3)	27 (18.5–49.5)	26 (16–42)	29 (21–57)	0.166
ALT, median (Q1-Q3)	20 (13-47.3)	19 (13-42.5)	25 (13.5–71)	0.192
Total Bilirubin, median (Q1-Q3)	0.9 (0.57–1.31)	0.88 (0.55–1.28)	0.91 (0.57–1.49)	0.950
Direct Bilirubin, median (Q1-Q3)	0.23 (0.13–0.4)	0.21 (0.13–0.39)	0.26 (0.12–0.47)	0.446
Lipase, median (Q1-Q3)	15 (8.8–27.5)	13 (6.5–27.5)	18 (11.5–31)	**0.043**
Lactate, median (Q1-Q3)	1.8 (1.4–2.2)	1.7 (1.4–2.2)	1.8 (1.5–2.3)	0.319
C-reactive protein, mean ± SD	208 ± 109	208 ± 112	209 ± 106	0.949
Procalcitonin, median (Q1-Q3)	0.71 (0.22–2.54)	0.51 (0.18–1.51)	1.57 (0.53–4.96)	**0.003**
Prognostic nutritional index, median (Q1-Q3)	37.5 (33.9–42.6)	37 (34-42.3)	38.5 (32.5–45.8)	0.401

*Fischer’s Exact test was used.

The lipase and procalcitonin levels of the patients at the time of first admission were significantly increased in patients with relapses (p = 0.043, p = 0.003). Except for procalcitonin, there was no statistical difference between groups, among the acute phase reactants given in Table 1. The median PNI value of the patients was 37.5 (p = 0.401). Lipase and procalcitonin were weakly associated with the recurrence of AC in correlation tests (lipase r = 0.202, p = 0.042; procalcitonin r = 0.296, p = 0.002).

The contents of the gallbladder were also grouped according to whether the stone was multiple or a single-large stone. There was a single-large stone in 36.3% of the patients, and multiple stones or a stone-biliary sludge complex in 63.7% of the patients. Although large stones were detected in 43.2% of the patients who had relapses, it was not statistically significant (p = 0.269). Likewise, the wall thickness, transverse diameter, longitudinal diameter, and the area of the gallbladder in the largest section were measured and compared. No significant difference was observed for these parameters (Table 2).

**Table 2:** Imaging and catheter-related features

	All patients	No recurrence	Recurrence	
	n = 102	n = 65	n = 37	p value
Presence of gallstones, n (%)				0.269
Single large stone	37 (36.3)	21 (32.3)	16 (43.2)	
Multiple	65 (63.7)	44 (67.7)	21 (56.8)	
Wall thickness (mm), median (Q1-Q3)	4.3 (3.5–5.5)	4.3 (3.5–5.8)	4.1 (3.5–5.3)	0.661
Gallbladder transverse diameter (mm), median (Q1-Q3)	44.5 (40.8–50)	44 (40.5–48.5)	45 (40.5–52)	0.512
Gallbladder longitudinal diameter (mm), median (Q1-Q3)	87.5 (76-102.3)	88 (75.5–102)	87 (76-102.5)	0.975
Gallbladder area (mm2), median (Q1-Q3)	3331 (2657–3737)	3329 (2539–3729)	3370 (2792–3843)	0.566
Choledochal diameter, n (%)				1.000*
Large	3 (2.9)	2 (3.1)	1 (2.7)	
Normal	99 (97.1)	63 (96.9)	36 (97.3)	
Scanning, n (%)				
Ultrasonography	88 (86.3)	55 (84.6)	33 (89.2)	0.519
Tomography	93 (91.2)	59 (90.8)	34 (91.9)	1.000*
MRCP	20 (19.6)	11 (16.9)	9 (24.3)	0.365
ERCP	2 (2)	1 (1.5)	1 (2.7)	1.000*
Tokyo classification, n (%)				0.257
Grade 2	73 (71.6)	49 (75.4)	24 (64.9)	
Grade 3	29 (28.4)	16 (24.6)	13 (35.1)	
Catheter insertion time, median (Q1-Q3)	2 (1–3)	2 (1–3)	2 (2–4)	**0.019**
Catheter insertion time, n (%)				**0.006**
In the first 24 h	40 (39.2)	32 (49.2)	8 (21.6)	
After 24 h	62 (60.8)	33 (50.8)	29 (78.4)	
Catheter drainage time, median (Q1-Q3)	32 (23–46)	32 (24–45)	36 (18.5–47)	0.824
Parenteral antibiotic duration, median (Q1-Q3)	7.5 (5–10)	7 (5–10)	9 (6–14)	0.067
Strong need for antibiotics, n (%)	27 (26.5)	14 (21.5)	13 (35.1)	0.135
Growth in bile culture, n (%)				0.313
There is reproduction	33 (32.4)	21 (32.3)	12 (32.4)	
No reproduction	25 (24.5)	19 (29.2)	6 (16.2)	
Not cultured	44 (43.1)	25 (38.5)	19 (51.4)	
Gallbladder content, n (%)				0.345*
Empyema	12 (11.8)	6 (9.2)	6 (16.2)	
Biliary	90 (88.2)	59 (90.8)	31 (83.8)	
Operation after extraction (days), median (Q1-Q3)	264 (43–462)	465 (404–602)	58 (26–112)	**< 0.001**
Operation after recurrence (days), median (Q1-Q3)	295 (76–510)	510 (456–672)	76 (69–152)	**< 0.001**
Cholecystectomy, n (%)	20 (19.6)	9 (13.8)	11 (29.7)	0.052
Type of operation, n (%)				0.065
Laparoscopic cholecystectomy	9 (8.8)	5 (7.7)	4 (10.8)	
Cholecystectomy from laparoscopic to open	6 (5.9)	4 (6.2)	2 (5.4)	
Open cholecystectomy	5 (4.9)	0	5 (13.5)	
Length of stay, median (Q1-Q3)	7.5 (5-11.3)	7 (5–10)	9 (6-14.5)	0.068
Catheter-related complication, n (%)	6 (5.9)	3 (4.6)	3 (8.1)	0.665*

*Fischer’s Exact test was used.

According to the TG18, 71.6% of the patients had grade 2 and 28.4% had grade 3 AC. There was no significant difference in the severity of AC between the two groups (p = 0.257). The time from the patient’s admission to the emergency service until the insertion of the catheter was longer in patients who had relapses (p = 0.019). A weak correlation was found between catheter insertion time and AC recurrence (r = 0.233, p = 0.018). Fewer recurrence was found in the patients who were inserted PC in the first 24 h after admission (p = 0.006). No difference was observed for recurrence between patients with > 2/≤2 week and > 1/≤1 month duration of drainage.

Although use of strong antibiotics was higher and the parenteral antibiotic therapy was longer in patients with recurrence, no significant difference was observed (p = 0.135, p = 0.067).

Bile cultures were obtained from 58 (56.9%) patients and growth was detected only 33 (56.9%) of them. The most common microorganisms were Escherichia Coli (30.3%), Enterococci (27.3%), Klebsiella Pneumoniae (18.2%) and Enterobacter (12.1%).

Gallbladder contents after catheterization were classified as purulent or bile. Although recurrence was higher in patients with purulent content this was not statistically significant (p = 0.345) (Table 2). The median recurrence time was 58 days. Recurrence-related mortality was 5.4% (Fig. 1).

When 37 patients with recurrence were evaluated, 59.5% of the patients had a recurrence within the first 3-months after catheter removal. Although it was observed that patients with recurrence in the first 3-months had more complaints of fever and accompanying pancreatitis at the time of admission, it was not statistically significant (p = 0.286, p = 0.505) (Table 3). Subgroup analysis was also performed for the effect of > 2/≤2 weeks and > 1/≤1 months of drainage time on recurrence in the first 3-months, but no significant difference was found between groups.

**Table 3:** Subgroup analysis according to recurrence time

	recurrence within 3 months	recurrence after 3 months	
	n = 22	n = 15	p value
Presence of fever, n (%)	9 (40.9)	3 (20)	0.286*
Pancreatitis, n (%)	2 (9.1)	0	0.505*
History of cholecystitis, n (%)	6 (27.3)	5 (33.3)	0.728*
Tokyo classification, n (%)			0.850
Grade 2	14 (63.6)	10 (66.7)	
Grade 3	8 (36.4)	5 (33.3)	
Grade at recurrence, n (%)			0.666
Grade 1	8 (36.4)	8 (53.3)	
Grade 2	11 (50)	5 (33.3)	
Grade 3	3 (13.6)	2 (13.3)	
Recurrence-related mortality, n (%)	2 (9.1)	0	0.505*
Catheter insertion time, median (Q1-Q3)	2 (1.8–4.5)	2 (2–3)	0.593
Catheter drainage time, median (Q1-Q3)	28 (14.5–42)	39 (29–70)	0.061
Lipase, median (Q1-Q3)	17.5 (10.5–29)	19 (12–40)	0.819
Procalcitonin, median (Q1-Q3)	1.41 (0.58–5.46)	1.74 (0.40–2.98)	0.772
Time to recurrence (days), median (Q1-Q3)	28.5 (18.8–57)	255 (140–424)	< 0.001

*Fischer’s Exact test was used.

**Fig. 2:**
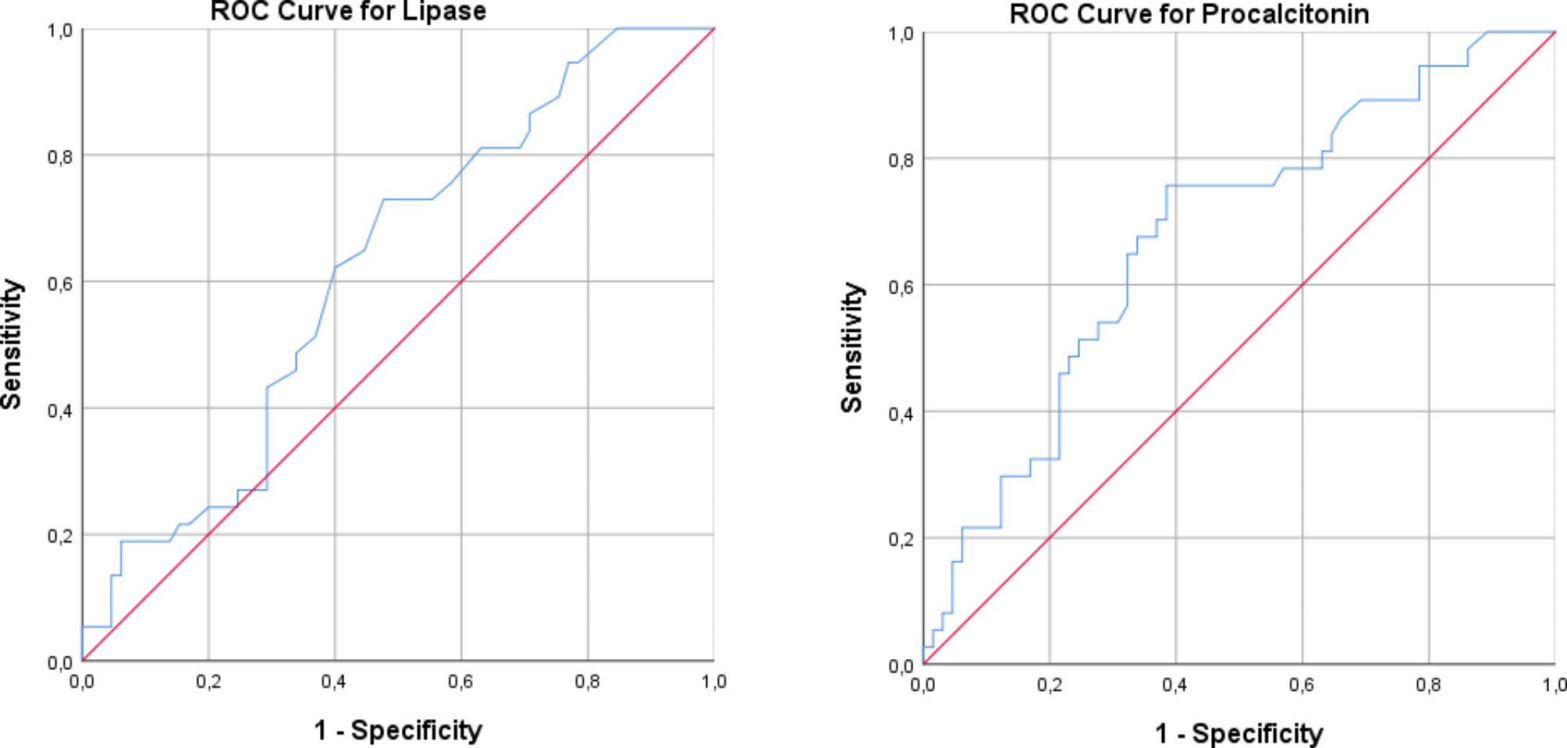
ROC analyzes for lipase and procalcitonin

The cut-off value for lipase parameter was 15.5 [sensitivity:62.2%; specificity:60%, AUC(95%CI): 0.621 (0.511–0.731), p = 0.043] and the cut-off value for procalcitonin parameter was 0.955 [sensitivity:67.6%; specificity:66.2%, AUC(95%CI): 0.678 (0.571–0.785), p = 0.003] (Fig. 2).

**Fig. 3:**
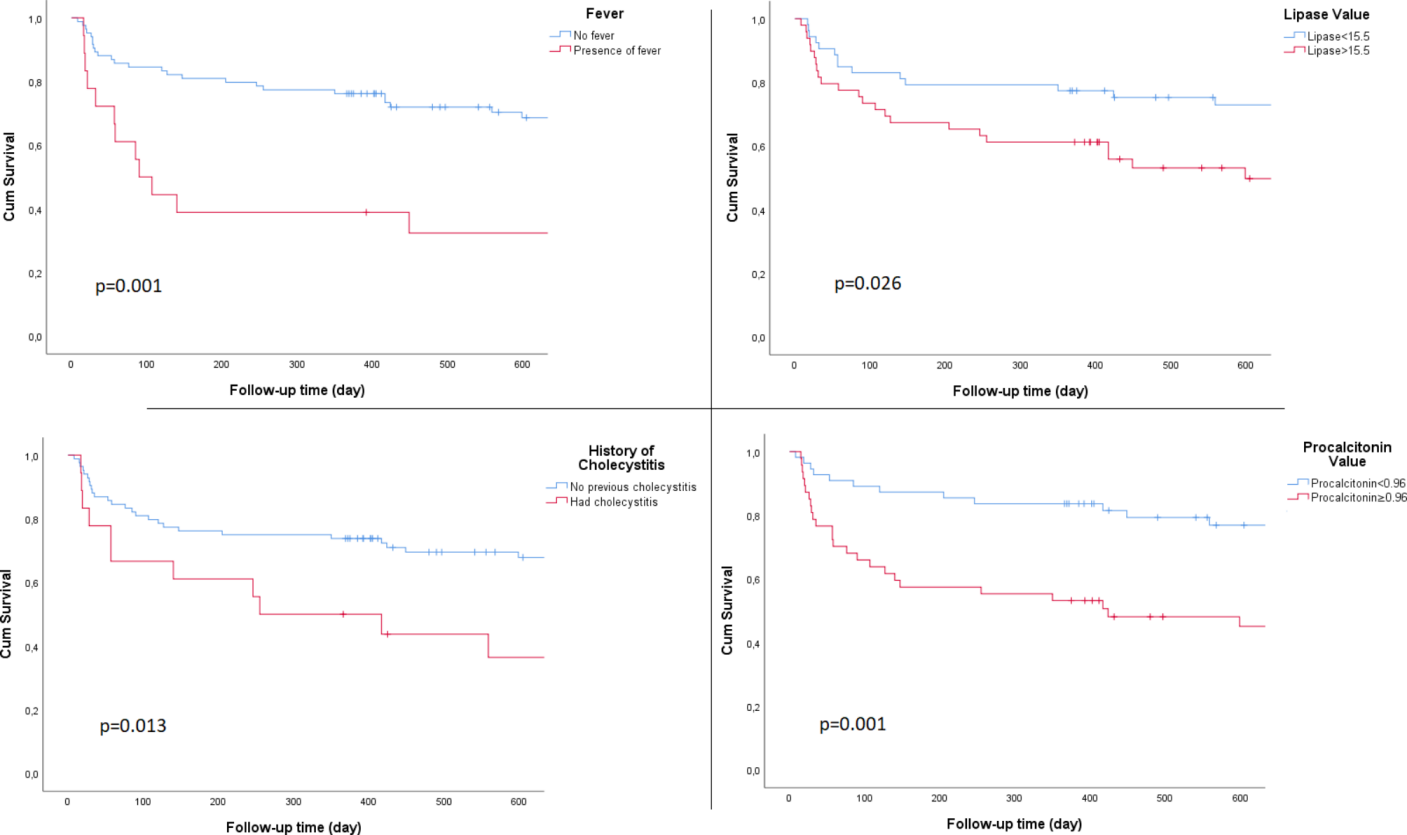
Recurrence-free survival graphs for fever, history of cholecystitis, procalcitonin and lipase value

Patients with no fever, no history of relaps, low lipase or low procalcitonin levels had a significantly longer recurrence-free survival (Fig. 3). Presence of fever, previous history of cholecystitis attack, lipase level > 15.5, and procalcitonin level ≥ 0.955 were risk factors for recurrence (Table 4).

**Table 4:** Univariate and multivariate analysis for recurrence-related risk factors

	Univariate analyses		Multivariate analyses	
	Odds ratio (95% CI)	p value	Odds ratio (95% CI)	p value
Presence of fever	**4.720 (1.593–13.981)**	**0.005**	**3.752 (1.045–13.468)**	**0.043**
Presence of history of cholecystitis, n (%)	**3.505 (1.221–10.063)**	**0.020**	**4.505 (1.171–17.325)**	**0.029**
Lipase elevation (> 15.5)	**2.464 (1.075–5.647)**	**0.033**	**3.586 (1.255–10.247)**	**0.017**
Procalcitonin elevation (≥ 0.96)	**4.072 (1.725–9.612)**	**0.001**	**6.044 (2.058–17.745)**	**0.001**
Catheter insertion time	1.148 (0.963–1.369)	0.123	1.165 (0.944–1.439)	0.154
Grade 3 acute cholecystitis	1.659 (0.688–3.999)	0.260	0.725 (0.236–2.229)	0.575
Presence of a single stone in the gallbladder	1.596 (0.694–3.671)	0.271	2.650 (0.916–7.671)	0.072
Purulent gallbladder contents	1.903 (0.566–6.397)	0.298	1.362 (0.303–6.123)	0.687
Gallbladder wall thickness	0.964 (0.742–1.251)	0.781	0.830 (0.599–1.150)	0.262

## Discussion

PC is a life-saving method with a minimally invasive intervention in the treatment of patients with AC. Although early cholecystectomy remains the standard treatment, perioperative mortality rates still remain high (up to 19%) in elderly or critically patients [4]. Therefore, PC is considered a safe alternative in surgically high-risk populations. PC is a very safe procedure with a low complication rate between 0 and 13% [10]. In our study, complication rate was 15.6%. As an experienced center, the predominance of surgical treatment in the treatment of AC and the referral of more complicated cases to PC may have increased the complication rate. In addition, it was thought that the complication rate might be higher than the literature, since the failure of catheterization and the development of acute abdomen after catheter removal were counted among the complications. Despite this, the absence of mortality due to PC procedure proves that the procedure is at a reliable level.

PC is a method often used as an interim treatment to prepare patients for interval cholecystectomy. However, in a study, it was reported that only 40% of the patients had cholecystectomy after PC [11]. In another study, the rate of elective cholecystectomy after catheterization was reported as 38.1% [4]. In our study, only 10.2% of the patients had the PC removal during surgery, and the rate of elective cholecystectomy following catheter extraction was 19.6%. This shows that PC can be a definitive treatment especially for patients with AC at high surgical risk. There are other studies advocating this in the literature [6, 12].

Various recurrence rates have been reported in the literature in patients who were followed up without surgery after removal of the PC. Li et al. reported the recurrence rate as 4.1% [6]. Wang et al. reported the recurrence rate as 6.5% in the first 2-months and 9.2% at 1-year [13]. Sanjay et al. reported the recurrence rate as 22%, and they reported that most of these patients were treated by reinserting a PC [5]. Park et al. reported the recurrence rate as 20.6%, while Garcia et al. reported the recurrence rate as 40% [2, 7]. In our study, the recurrence rate of AC was 36.3%. According to some studies in the literature, the recurrence rate ranged from 4 to 40%. The severity of recurrence is classified according to the TG18, and it is thought that the presence of mild cholecystitis increases the recurrence rate.

The recurrence rate in the first 3-months was 21.6%, 3-month-1 year was 8.8%, and after 1-year was 5.9%. Wang et al. also reported that recurrence was more common in the first 2-months [13]. It has been reported in animal experiments that irritation of the gallbladder mucosa predisposes to AC [14]. In addition, the theory of bacterial colonization secondary to the inserted catheter may help explain that recurrence is more common in the first 3-months after the first AC attack. However, there is insufficient evidence to elucidate longer-term relapses.

Various studies have been conducted to determine the parameters associated with recurrence. Chang et al. [12] and Garcia et al. [7] could not detect any factor related to low recurrence rate. Bergman et al. reported higher recurrence rates in males [15]. However, this study had differences compared to our study, such as the inclusion of patients who underwent ERCP other than cholecystostomy and who also had biliary pathologies other than the gallbladder. Hsieh et al. reported prolonged drainage time (> 2 weeks) and high C-reactive protein (CRP) (> 15 mg/dl) levels as factors predicting recurrence [16]. Wang et al. reported that patients with complicated cholecystitis, long-term need for parenteral antibiotics (> 10 days), high WBC (≥ 18,000/µL), or requiring long-term PC drainage (> 32 days) are more likely to recur [13]. In our study, none of these previously examined parameters such as age, gender, comorbidities, CCI, drainage time, parenteral antibiotic duration, WBC and CRP were associated with recurrence.

In the parameters measured in this study, previous history of cholecystitis attack, fever symptom at presentation, and elevated lipase (> 15.5) and procalcitonin (≥ 0.96) were associated with recurrence. Also these four parameters were detected as risk factors for recurrence.

The duration of drainage time has not been clearly determined in the literature. In our study subgroup analyzes were performed as > 2/≤2 weeks and > 1/≤1 months, but no significant difference was found between them. We think that the time of PC removal should be decided according to the clinical condition of the patient and the amount of drainage. Studies state the rate of positive bile culture between 16 and 80% [17–19]. In our study, bile culture positivity was 56.9%.

Ha et al. reported 1-year and 3-year recurrence of AC as 35% and 46%, respectively [20]. In addition, they determined that the stone size in the gallbladder is ≥ 1 cm as a risk factor for recurrence [20]. In our study, gallstones were grouped as having a single-large stone or multiple bile-sludge complexes, regardless of size. Although the presence of single-large stone was found to be higher in those with recurrent cholecystitis attacks, this relationship was not significant. The relationship could not be detected due to the measurements were not sufficiently specific and the sizes of the stones were not measured.

Early cholecystostomy catheter placement prevents adhesion formation and severe fibrosis [21]. There are studies in the literature examining the relationship between cholecystostomy catheter insertion time and short-term outcomes. Üstüner et al. [21] and Zazour et al. [22] could not find any relationship between early (first 24-hours) or late (after 24-hours) catheter insertion time with morbidity and mortality. Bikel et al. showed that early (first 2-days) insertion of the catheter reduces the rate of conversion to open surgery [23]. Chou et al. showed that insertion of a catheter in the first 24-hours shortens the hospital stay [24]. In our study we determined that the duration of catheter insertion was longer in patients with relapses. Also fewer recurrence was detected in the patients who were inserted PC in the first 24-hours. Although the duration of insertion was associated with recurrence, the duration of catheter insertion was not a risk factor for recurrence. In addition, grade 3 AC, purulent gallbladder content and gallbladder wall thickness were not associated with recurrence.

There are some limitations of this study. First, the retrospective nature of the study may have caused losses in the 1-year follow-up of the patients. Second, we cannot exclude the possibility that patients in our study were treated for relapse at other hospitals. However, since all patients present to our emergency department at the onset of AC, it suggests that many may benefit from the same emergency medical services in case of relapse. This can compensate for the underestimation to some extent.

## Conclusion

Percutaneous cholecystostomy is an effective treatment method in patients with acute cholecystitis at high surgical risk. Insertion of the catheter within the first 24 h may reduce the frequency of recurrence. Recurrence is more common in the first 3 months following removal of the PC. Having a previous history of cholecystitis, fever symptom at the time of admission, elevated lipase and procalcitonin are risk factors for recurrence.

## Data Availability

The datasets used and/or analyzed during the current study are available from the corresponding author on reasonable request.
